# Supplementary material for: The effects of 17α-estradiol treatment on endocrine system revealed by single-nucleus transcriptomic sequencing of hypothalamus
Source: eLife. 2025 Sep 25;13:RP100346. doi: 10.7554/eLife.100346 (PMC12463393; doi:10.7554/eLife.100346)
Supplement: MDAR checklist [file elife-100346-mdarchecklist1.docx]

**Materials Design Analysis Reporting (MDAR)**

**Checklist for Authors**

The [MDAR framework](https://osf.io/xfpn4/) establishes a minimum set of requirements in transparent reporting mainly applicable to studies in the life sciences.

*eLife* asks authors to **provide detailed information within their article** to facilitate the interpretation and replication of their work. Authors can also upload supporting materials to comply with relevant reporting guidelines for health-related research (see [EQUATOR Network](http://www.equator-network.org/%20)), life science research (see the [BioSharing Information Resource](http://biosharing.org/)), or animal research (see the [ARRIVE Guidelines](http://www.plosbiology.org/article/info:doi/10.1371/journal.pbio.1000412) and the [STRANGE Framework](https://doi.org/10.1038/d41586-020-01751-5); for details, see *eLife*’s [Journal Policies](https://reviewer.elifesciences.org/author-guide/journal-policies)). Where applicable, authors should refer to any relevant reporting standards materials in this form.

For all that apply, please note **where in the article** the information is provided. Please note that we also collect information about data availability and ethics in the submission form.

**Materials:**

| **Newly created materials** | **Indicate where provided: section/figure legend** | **N/A** |
| --- | --- | --- |
| The manuscript includes a dedicated "materials availability statement" providing transparent disclosure about availability of newly created materials including details on how materials can be accessed and describing any restrictions on access. | Materials and methods section | Not applicable, no newly created materials in this study |
|  |  |  |
| **Antibodies** | **Indicate where provided: section/figure legend** | **N/A** |
| For commercial reagents, provide supplier name, catalogue number and [RRID](https://scicrunch.org/resources), if available. | N/A | N/A |
|  |  |  |
| **DNA and RNA sequences** | **Indicate where provided: section/figure legend** | **N/A** |
| Short novel DNA or RNA including primers, probes: Sequences should be included or deposited in a public repository. | Materials and methods section | All sequencing data are available at GEO accession number GSE248413. |
|  |  |  |
| **Cell materials** | **Indicate where provided: section/figure legend** | **N/A** |
| Cell lines: Provide species information, strain. Provide accession number in repository OR supplier name, catalog number, clone number, OR RRID. | N/A | N/A |
| Primary cultures: Provide species, strain, sex of origin, genetic modification status. | N/A | N/A |
|  |  |  |
| **Experimental animals** | **Indicate where provided: section/figure legend** | **N/A** |
| Laboratory animals or Model organisms: Provide species, strain, sex, age, genetic modification status. Provide accession number in repository OR supplier name, catalog number, clone number, OR RRID. | Materials and methods section (Animals, treatment and tissues subsection). | Norway brown male rats (12 months old and 1-month old) were acquired from Charles River (Beijing). |
| Animal observed in or captured from the field: Provide species, sex, and age where possible. | N/A | N/A |
|  |  |  |
| **Plants and microbes** | **Indicate where provided: section/figure legend** | **N/A** |
| Plants: provide species and strain, ecotype and cultivar where relevant, unique accession number if available, and source (including location for collected wild specimens). | N/A | N/A |
| Microbes: provide species and strain, unique accession number if available, and source. | N/A | N/A |
|  |  |  |
| **Human research participants** | **Indicate where provided: section/figure legend) or state if these demographics were not collected** | **N/A** |
| If collected and within the bounds of privacy constraints report on age, sex, gender and ethnicity for all study participants. | N/A | N/A |

**Design:**

| **Study protocol** | **Indicate where provided: section/figure legend** | **N/A** |
| --- | --- | --- |
| If the study protocol has been pre-registered, provide DOI. For clinical trials, provide the trial registration number OR cite DOI. | N/A |  |
|  |  |  |
| **Laboratory protocol** | **Indicate where provided: section/figure legend** | **N/A** |
| Provide DOI OR other citation details if detailed step-by-step protocols are available. | N/A |  |
|  |  |  |
| **Experimental study design (statistics details) *** | | |
| **For in vivo studies: State whether and how the following have been done** | **Indicate where provided: section/figure legend. If it could have been done, but was not, write “not done”** | **N/A** |
| Sample size determination | Materials and methods section (Animals, treatment and tissues subsection) | No formal sample size calculation was performed (not done). The sample size was determined based on preliminary experiments with 4 rats per group (O group, O.T group, Y group) sufficient to support the statistical power of snRNA-seq and hormone detection. |
| Randomisation | Materials and methods section (Animals, treatment and tissues subsection) | Aged rats were randomly allocated into control and 17α-estradiol-treated groups. |
| Blinding | Materials and methods section | Blinding was not implemented (not done). Since experimental groups were distinguished by hormone treatment (identifiable via treatment protocols), and data collection (e.g., serum detection, HE staining) had no subjective bias, blinding design was not conducted. |
| Inclusion/exclusion criteria | Materials and methods section and Figure legends | No pre-established inclusion/exclusion criteria for animals; healthy Norway brown male rats were selected based on the study design. |
|  |  |  |
| **Sample definition and in-laboratory replication** | **Indicate where provided: section/figure legend** | **N/A** |
| State number of times the experiment was replicated in the laboratory. | Materials and methods section (Enzyme immunoassays, snRNA-seq data processing subsection) and Figure legends (e.g., Figure 5E, 7B) | Biological replicates: At the animal level - 4 independent rats per group; For snRNA-seq - 2 hypothalamic sample pools in O group, 3 sample pools each in Y and O.T groups (each pool from 1 rat); For hormone detection (ELISA) - serum from 3 rats per group, with 2 technical replicates per sample. |
| Define whether data describe technical or biological replicates. | Materials and methods section and Figure legends | Biological replicates include independent rats per group and hypothalamic sample pools from individual rats; Technical replicates refer to 2 repeated detections of each serum sample in ELISA. |
|  |  |  |
| **Ethics** | **Indicate where provided: section/submission form** | **N/A** |
| Studies involving human participants: State details of authority granting ethics approval (IRB or equivalent committee(s), provide reference number for approval. | **N/A** | **N/A** |
| Studies involving experimental animals: State details of authority granting ethics approval (IRB or equivalent committee(s), provide reference number for approval. | Materials and methods section (Animals, treatment and tissues subsection) | All animal procedures were reviewed and approved by the Institutional Animal Care and Use Committee at Nantong University (approval number: S20210225-012). |
| Studies involving specimen and field samples: State if relevant permits obtained, provide details of authority approving study; if none were required, explain why. | **N/A** | **N/A** |
|  |  |  |
| **Dual Use Research of Concern (DURC)** | **Indicate where provided: section/submission form** | **N/A** |
| If study is subject to dual use research of concern regulations, state the authority granting approval and reference number for the regulatory approval. | **N/A** | **N/A** |

**Analysis:**

| **Attrition** | **Indicate where provided: section/figure legend** | **N/A** |
| --- | --- | --- |
| Describe whether exclusion criteria were pre-established. Report if sample or data points were omitted from analysis. If yes, report if this was due to attrition or intentional exclusion and provide justification. | Materials and methods section (Statistics subsection) | No pre-established exclusion criteria were set. No samples or data points were omitted from the analysis (no attrition or intentional exclusion). |
|  |  |  |
| **Statistics** | **Indicate where provided: section/figure legend** | **N/A** |
| Describe statistical tests used and justify choice of tests. | 1. Materials and methods section (Statistics subsection) | 1. Pearson correlation coefficient: Analyzes linear relationships (e.g., OXPHOS subunits vs. pathways, TFs vs. signaling networks); p < 0.05. Ideal for continuous variables to quantify co-expression reliably. 2. Fast Wilcoxon rank sum test: Compares O/O.T/Y groups for snRNA-seq data (e.g., gene expression, cell proportions); ≥15 cells/group. Non-parametric, avoids normality assumptions for skewed single-cell data. 3. auROC analysis: Evaluates discriminative ability of signatures/pathways (e.g., top synaptic pathways). Intuitive AUC ranking identifies key group-distinguishing features. 4. FindMarkers (bimod test): Identifies DEGs (e.g., O.T vs. O in Crh neurons); filters: min.pct=0.1, logfc=0.25. Suits snRNA-seq’s bimodal "on/off" expression to detect meaningful differences. 5. WebGestalt (GO enrichment): Enriches DEGs for GO terms; FDR < 0.05. Reduces false positives via FDR correction, validates DEG biological relevance. 6. Univariate Linear Model (ulm, decoupleR): Scores TF/pathway activity (e.g., top 25 TFs in Oxt neurons). Standardizes quantification for cross-group comparison. 7. Augur (cell prioritization): Prioritizes responsive neuron subtypes (e.g., top 20 neuropeptide-secreting subtypes); ≥6 cells/subcluster. AUC-based, optimized for snRNA-seq to focus on key cells. 8. MR methods (IVW/MR Egger): Analyzes OXT/GNRH1-endocrine trait causality; excludes weak instruments (F<10); IVW p<0.05. Multi-methods ensure robustness (IVW for efficiency, MR Egger for pleiotropy). 9. Two-tailed unpaired t-test: Compares serum/plasma hormone levels (e.g., Crh, testosterone). Suits normally distributed, independent samples for simple 2-group comparison. 10. Harmony (batch correction): Removes snRNA-seq technical variation. Ensures group differences reflect biology, not artifacts. 11. Descriptive stats (mean/SD): Summarizes data (e.g., testis inflammation index, pathway expression). Clarifies central tendency/variability for easy interpretation. |
|  |  |  |
| **Data availability** | **Indicate where provided: section/submission form** | **N/A** |
| For newly created and reused datasets, the manuscript includes a data availability statement that provides details for access (or notes restrictions on access). | Materials and methods section | All sequencing data are available at GEO accession number GSE248413. |
| When newly created datasets are publicly available, provide accession number in repository OR DOI and licensing details where available. | Materials and methods section | GEO accession number GSE248413 |
| If reused data is publicly available provide accession number in repository OR DOI, OR URL, OR citation. | **N/A** |  |
|  |  |  |
| **Code availability** | **Indicate where provided: section/figure legend** | **N/A** |
| For any computer code/software/mathematical algorithms essential for replicating the main findings of the study, whether newly generated or re-used, the manuscript includes a data availability statement that provides details for access or notes restrictions. | **N/A** | **N/A** |
| Where newly generated code is publicly available, provide accession number in repository, OR DOI OR URL and licensing details where available. State any restrictions on code availability or accessibility. | **N/A** | **N/A** |
| If reused code is publicly available provide accession number in repository OR DOI OR URL, OR citation. | **N/A** | **N/A** |

**Reporting:**

The MDAR framework recommends adoption of discipline-specific guidelines, established and endorsed through community initiatives.

| **Adherence to community standards** | **Indicate where provided: section/figure legend** | | **N/A** |
| --- | --- | --- | --- |
| State if relevant guidelines (e.g., ICMJE, MIBBI, ARRIVE, STRANGE) have been followed, and whether a checklist (e.g., CONSORT, PRISMA, ARRIVE) is provided with the manuscript. | Materials and methods section and Supporting materials | **Adherence to relevant guidelines**:   - 1. **ARRIVE guidelines (Animal Research: Reporting of In Vivo Experiments)**: Followed. Details include clear description of animal sources (Charles River, Beijing), grouping (O, O.T, Y) and treatment (17α-estradiol dose, duration), sample collection (hypothalami, testes, serum) with standardization (blood collected 9:00–9:30 a.m. to minimize hormone fluctuation), euthanasia method (CO₂), and ethical approval (Institutional Animal Care and Use Committee of Nantong University, approval number: S20210225-012)—all align with ARRIVE requirements for transparent reporting of animal experiments.   2. **MIBBI (Minimum Information for Biological and Biomedical Investigations)**: Partially followed. Key experimental details are reported, including reagent sources (e.g., ELISA kits from Raybiotech, Enzyme-linked Biotechnology), snRNA-seq protocols (10X Chromium platform, Illumina HiSeq™ 4000 sequencing), and data analysis tools (Cell Ranger, Seurat, TwoSampleMR)—meeting MIBBI’s minimum information standards for reproducibility.   3. **Other guidelines (ICMJE, STRANGE, CONSORT, PRISMA)**: Not applicable. ICMJE focuses on clinical trial authorship/disclosure, STRANGE on study sample representativeness (less relevant for controlled animal studies), and CONSORT/PRISMA on randomized trials/systematic reviews—none match the study’s animal-based and omics research design. | |

* We provide the following guidance regarding transparent reporting and statistics; we also refer authors to [Ten common statistical mistakes to watch out for when writing or reviewing a manuscript](https://doi.org/10.7554/eLife.48175).

**Sample-size estimation**

- You should state whether an appropriate sample size was computed when the study was being designed
- You should state the statistical method of sample size computation and any required assumptions
- If no explicit power analysis was used, you should describe how you decided what sample (replicate) size (number) to use

**Replicates**

- You should report how often each experiment was performed
- You should include a definition of biological versus technical replication
- The data obtained should be provided and sufficient information should be provided to indicate the number of independent biological and/or technical replicates
- If you encountered any outliers, you should describe how these were handled
- Criteria for exclusion/inclusion of data should be clearly stated
- High-throughput sequence data should be uploaded before submission, with a private link for reviewers provided (these are available from both GEO and ArrayExpress)

**Statistical reporting**

- Statistical analysis methods should be described and justified
- Raw data should be presented in figures whenever informative to do so (typically when N per group is less than 10)
- For each experiment, you should identify the statistical tests used, exact values of N, definitions of center, methods of multiple test correction, and dispersion and precision measures (e.g., mean, median, SD, SEM, confidence intervals; and, for the major substantive results, a measure of effect size (e.g., Pearson's r, Cohen's d)
- Report exact p-values wherever possible alongside the summary statistics and 95% confidence intervals. These should be reported for all key questions and not only when the p-value is less than 0.05.

**Group allocation**

- Indicate how samples were allocated into experimental groups (in the case of clinical studies, please specify allocation to treatment method); if randomization was used, please also state if restricted randomization was applied
- Indicate if masking was used during group allocation, data collection and/or data analysis
